# The Assessment of Landscape Expressivity: A Free Choice Profiling Approach

**DOI:** 10.1371/journal.pone.0169507

**Published:** 2017-01-23

**Authors:** Stephan P. Harding, Sebastian E. Burch, Françoise Wemelsfelder

**Affiliations:** 1 Schumacher College, Dartington, Devon, United Kingdom; 2 Posada del Valle, Collía, Arriondas, Asturias, Spain; 3 Animal and Veterinary Sciences Group, Scotland’s Rural College, Roslin Institute Building, Easter Bush, United Kingdom; Fondazione Edmund Mach Centro Ricerca e Innovazione, ITALY

## Abstract

In this paper we explore a relational understanding of landscape qualities. We asked three independent groups of human observers to assess the expressive qualities of a range of landscapes in the UK and in Spain, either by means of personal visits or from a projected digital image. We employed a Free Choice Profiling (FCP) methodology, in which observers generated their own descriptive terminologies and then used these to quantify perceived landscape qualities on visual analogue scales. Data were analysed using Generalised Procrustes Analysis, a multivariate statistical technique that does not rely on fixed variables to identify underlying dimensions of assessment. The three observer groups each showed significant agreement, and generated two main consensus dimensions that suggested landscape ‘health’ and ‘development in time’ as common perceived themes of landscape expressivity. We critically discuss these outcomes in context of the landscape assessment literature, and suggest ways forward for further development and research.

## Introduction

A perennial concern in scientific landscape assessment is whether qualities we ascribe to landscapes and their features such as meadows, woodlands and mountains are an intrinsic, objective aspect of the landscape itself, or reside only in the eye of the beholder as an aesthetic, subjective perception [1]. Both such objectivist and subjectivist stances have inspired a large variety of landscape studies in recent times. However, a core feature which these studies tend to have in common is that only humans are considered to be psychologically engaged with landscapes–other sentient organisms play no role. On the one hand, subjectivist approaches tend to address landscape qualities as constructs ‘in the mind’ formed through socio-cultural conditioning and individual imagination. In this perspective landscapes hardly feature other than as an adjunct to human experience. On the other hand, approaches that ascribe some kind of objective standing to landscapes tend to do so in functional ecological terms, focusing on features amenable to quantitative measurement, such as number of hedgerows, acres of woodland, or number and orientation of fields. The term ‘objective’ here refers to locating qualities ‘out there’ in the landscape, while physicalising these qualities in externalist, mechanistic terms, that dismantle and deflate any psychological significance qualities may carry through the activities of sentient organisms. Thus neither subjectivist nor objectivist approaches tend to consider landscape itself as bearing meaning–its meaning is thought to be purely symbolic in terms of what it affords human society [2].

In recent years much emphasis has been placed on the complexity of landscapes and the need for transdisciplinary collaboration to capture a landscape’s multi-layered eco-social feedback loops [3–6]. However, such systems-based approaches tend to preserve the language typically used in subjectivist or objectivist methods, and so preserve the human-centred perspective inherent in these methods. Such approaches may be called holistic in that they acknowledge the greater whole of complex landscape systems, but they tend not to attribute inherent meaning to these systems, other than perhaps indirectly when describing them, for example, as ‘self-organising’ [3, 7].

In this paper we wish to explore a relational understanding in which landscape is considered *part of* the communication taking place between living beings, not merely a substrate *for* it. Such an approach has objectivist aspects in that it considers landscape qualities to be real,–and includes subjectivist aspects in recognising that this reality, *as* communication, requires human engagement. However it aims to reach beyond the subject/object, inside/outside dichotomy, to better reflect the dynamics of how sentient organisms actually live, through continuously responsive, expressive communication with all that surrounds them [8–10]. Historically the very term ‘landscape’ already reflects an intention to physicalise and contain natural organisation, as if plants and animals in their habitats collectively are nothing but a physical territory [4]. But there are alternative formulations. Following indigenous hunter-gatherers, anthropologist Tim Ingold [11,12] proposes we might understand landscape not as a terrain to live *in* or *on*, but as a dynamic process of living *with*, a ‘dwelling together’ of natural entities in continuous co-development and co-formation. Anthropologist David Anderson [13] describes indigenous people’s practice of such ‘living with’ nature as ‘sentient ecology’. Other pioneering thinkers on the participative nature of our life-world are Arne Naess [14], David Abram [15], and Margaret Colquhoun [16], to name but a few. These writers all argue in various ways that a landscape’s expressive qualities are as primary and natural as its physical quantities, and should be given serious academic consideration. This is not, it should be noted, the same as granting landscapes individual agency and emotional experience as is done with humans and animals. But it is to recognise that psychological relationship emerges not merely *within* us, but *with* us, *around* us, in how acting and responding organisms collectively create and perceive landscape [15,17]. As landscape architect and planner Anne Whiston Spirn [18] puts it in her book ‘The Language of Landscape’: “Landscape has meaning. … Significance is there to be discovered, inherent and ascribed, shaped by what senses perceive, what instinct and experience read as significant, what minds know. Any organism with senses has the potential to read and understand landscape” (p.18).

In this study we explore what happens if human observers are asked to engage with different landscapes and to assess the expressive qualities they perceive to be communicated. We did this three times with three independent groups of people: two groups describing the same 10 Devon landscapes either by means of personal visits or from a projected digital image, and one group describing 11 Spanish landscapes through personal visits. It was clearly essential not to bias these observers in their interpretations, and so we employed a Free Choice Profiling (FCP) methodology, which is specifically designed to let observers generate their own descriptive terminologies which they then use for further assessment. FCP originated in food sensory science, to allow consumer panels to assess food qualities in their own words [19]. However, it has since also been developed to explore expressive qualities of animals in studies of animal health and welfare [20,10]. These studies have established that animal qualities can be reliably addressed and investigated, and that they have scientific validity in helping to interpret physical measures of animal health and welfare [21,22]. Thus FCP seems a potentially valuable tool to enable the study of emergent qualities in different fields of interest. In this paper, we explore the application of FCP to landscape studies, and consider the relevance this may have for the development of ecological sustainability.

## Methods

### Landscapes

For the Devon outdoor study (DOS), we selected 10 landscapes, and 10 viewpoints from which to view these landscapes, in the Dartmoor region of Devon County, UK. This included a diversity of habitats with varying degrees of human interference, ranging from an agricultural field and a man-made reservoir, to open moor- and heath land and riverside forest in the valley of the river Dart. The Devon indoor study (DIS) used digital images of these same 10 landscapes, taken with an Olympus C765UZ digital camera from the exact same viewpoints as visited for DOS. These images were projected on to a 198 x 166 cm manual projection screen using a digital powerpoint projector. For the Asturias study (AS), we selected 11 landscapes in the Asturian region of Spain, also including a diversity of habitats with varying degrees of human interference, ranging from a eucalyptus plantation and grazing pastures to various mountain locations. Tables 1 and 2 present brief descriptions of these visited landscapes.

**Table 1:** Brief description of Devon landscapes.

Devon site number	Location	Brief landscape description
1	Riverside, Hembury Woods	Fast, wild river flowing over many small boulders with occasional white water, surrounded by extensive mature native woodland.
2	Oak woodland, Hembury Woods	A mostly even-aged stand of tall oak woodland about 40 years old. Well-developed shrub and herb layers consisting of native species.
3	Heathland, Hembury Woods	Heavily grazed open areas of low grass, surrounded by abundant gorse bushes and scattered small native trees.
4	Venford high moor, Dartmoor	Expansive vista of open, mostly treeless moorland with low undulating hills stretching towards the horizon.
5	River Dart valley from Bench Tor, Dartmoor	Deeply incised, steep river valley completely forested with mature native woodland. Glimpses of the river visible as turbulent flowing white water.
6	Bench Tor Rocks, Dartmoor	Long low granite outcrop (tor) with many large, deeply weathered cracks with grass, and a few low trees growing on top.
7	View from Combstone Tor, Dartmoor	Expansive views of an extensive patchwork of fields surrounded by hedgerows interlaced with ribbons of native woodland.
8	Venford Reservoir, Dartmoor	Large reservoir dominating the foreground, surrounded by mature pine trees in the middle distance and expansive open moor land beyond.
9	Venford Brook, Dartmoor	Small, turbulent stream valley flowing through relatively undisturbed native woodland. Several small waterfalls. Abundant moss-covered granite boulders. Ground layer of bracken, wood sorrel and other native plants.
10	Field, Dartington Hall Estate	Large, open field planted with low grass cut in rows. Mature native woodlands in the far distance.

**Table 2:** Brief description of Asturias landscapes.

Asturias site number	Location	Brief landscape description
1	West side of Collía mountain	Part of a relatively low, long mountain range. Outcrops of limestone, with gorse, heather and grass. Mostly deforested. Heavily grazed, and occasionally burnt.
2	Closed wooded meadow	A small enclosed pasture, surrounded by a tall natural hedge of mixed native trees and shrubs.
3	Open meadow	A relatively small field with occasional small outcrops of limestone and isolated trees.
4	Vegetable garden	A large fenced vegetable plot by the side of a house.
5	Ornamental garden	An extensive area of ornamental flowers, shrubs and trees. Many pollard fruit trees.
6	Sueve mountain range	A long, low mountain range overlooking an expansive valley.
7	Eucalyptus plantation	An intensive plantation of pole-stage Eucalyptus trees around 15m high.
8	La Forcada mountain	A small limestone mountain with occasional houses and patches of native mixed woodland at the base.
9	East side of Collía mountain	Extensive limestone outcrops with gorse, heather and grass adjoining patches of eucalyptus and pastures.
10	Razed mountain	One of many small deforested hills with a wide track excavated to the top. Occasional planted pine trees.
11	Collía village	An overview of a small village of around 20 old and new houses, surrounded by low hills and mature woodland.

### Observers

For DOS we recruited 10 students participating in the MSc in Holistic Science at Schumacher College in Devon, UK. These students varied in age, gender (5 male, 5 female) and nationality, and were all proficient in the English language. For DIS we recruited 10 short course participants at Schumacher College (all different from DOS observers), also of varying age, gender (4 male, 6 female) and nationality, all proficient in English language. For AS 14 observers were recruited of varying age and gender (5 male, 9 female), with 9 native Spanish and 5 native English speakers.

Observers gave verbal informed consent to participate in this study. Written consent was not deemed necessary since observers were not required to reveal personal information that might have compromised them in any way. The study procedures were carefully explained to prospective observers, who gave their verbal consent upon deciding to take part. Their participation thus constitutes a record of their consent. The Ethics committee at Schumacher College approved this consent process and also specifically approved this study.

### Experimental Procedures

Observers were instructed to assess the expressive qualities of Devon and Asturian landscapes using a Free Choice Profiling (FCP) methodology, following procedures developed by Wemelsfelder and colleagues [20]. FCP consists of two phases. In phase 1 observers focus on generating their own personal descriptors for landscape quality, either while visiting landscapes (DOS) or while looking at photographs (DIS). In phase 2 these descriptors are fitted with visual analogue scales (VAS), and observers revisit observed landscapes/photos and use their personal rating scales to quantitatively score the landscapes’ expressive qualities.

#### FCP phase 1

All three studies were initiated by an hour’s instruction for participating observers, gathered together in either Devon or Asturias. Observers in one study were not aware of the existence of the other studies. Observers were informed that the objective of the study was to explore people’s spontaneous qualitative assessments of landscape, using a novel FCP methodology. We explained that the focus in this study was on the perceived expressive character of the landscapes, not on their own response to this perceived character. We acknowledged that as part of the communicative process observers were likely to have an immediate response to the different landscapes, but we emphasized that we wanted observers to focus on the qualities emanating from the landscape itself rather than on their own response. For example, one could perceive a dark cave as ‘overwhelming’ or ‘claustrophobic’, but these terms describe effects on the observer rather than qualities of the cave itself, which might be characterised as ‘expansive’ or ‘still’. The difference between these two approaches is of course not always sharp—sometimes the terms for a landscape’s expression and one’s response can be the same—an open landscape can make one feel open. Having clarified these issues, we then discussed FCP procedures with observers and what they were expected to see and do in each of its two phases.

Observers were told that in phase 1 they would be taken to visit, or shown images of, 10 (Devon) or 11 (Asturian) pre-selected landscape viewing points, and that at each of these points, or after viewing each of these photographs, they should find and write down descriptors that for them captured the expressive qualities of the observed landscape. We explained that with each new landscape they were entirely free to choose new terms or re-iterate previously used terms, as long as they chose the best terms for each individual landscape. We asked observers to avoid terms describing a landscape’s physical traits, such as ‘damp’ or ‘rocky’, except when a term also had a more psychological connotation, such as for example in ‘rough’, or ‘spacious’. Observers were asked to refrain from discussing their terms with others throughout the day to ensure independence of their assessments.

Having been given this introduction, observers in DOS and AS were then taken by minibus along a pre-determined route visiting the 10 or 11 selected landscape viewing points. At each point they found a comfortable place to sit or stand and for 5 minutes directed their attention towards the landscape in front of them. To enhance a common focus the leader of each study outlined to observers the spatial boundaries within which they should watch the landscape. At the 5 minute signal observers were given another 5 minutes to identify suitable descriptors and write these down on provided forms. Native Spanish speakers in Asturias gave their terms in Spanish. For the benefit of this paper these terms were later translated into English by one of the authors (translations available in S4 File).

In DIS, the 10 observers stayed in the room where they had been given instructions, and were shown a PowerPoint presentation consisting of digital images of the same 10 Devon landscapes selected for the Devon outdoor study. These photographs were taken from within the same spatial boundaries as outlined to observers in the outdoor study, and shown in the same order as the one in which the landscapes were visited during the outdoor study. Each landscape was projected on screen for 2 minutes, and observers were given another 2 minutes to write down their terms describing the perceived expressive qualities of that landscape.

Thus at the end of Phase 1, each observer in each of the three studies had compiled a personal set of terms describing the expressive qualities of the 10 or 11 visited/viewed landscapes.

#### FCP phase 2

To prepare for phase 2, for each individual observer their personal terms were printed on a form next to visual analogue scales (VAS) of 125 millimetre length ranging from ‘minimum’ (quality absent) to ‘maximum’ (quality could not be more dominant). Terms were presented in random order but avoiding proximity of similar terms, and also avoiding clustering of apparently positively and negatively valenced terms. Because the quantitative scoring of terms with negative pre-fixes such as un-, in-, or non- easily creates confusion, such terms were put on the form in their positive version (e.g. ‘un-harmonious’ became ‘harmonious’), except in those cases where the negative version is more commonly used than the positive one (e.g. ‘unshakable’).

In all three studies Phase 2 took place one week after Phase 1. The same observer groups were taken to the same Devon and Asturian landscapes, but in a different sequence than in Phase 1. In DIS observers were presented with the same images as in Phase 1, again following the order of DOS in this phase. Observers again for 5 minutes (or for 2 minutes in DIS) directed their attention towards the landscape in front of them, and after a signal scored the expressive qualities of that landscape on each of their terms by marking the VAS at the appropriate point between ‘minimum’ and ‘maximum’.

## Statistical Analysis

### Data Processing

The scores attributed by observers to landscape qualities were determined by measuring the distance in millimeters between the left ‘minimum’ point of the VAS and the point where the observer ticked the line. These scores were entered into data matrices, one for each observer, with each matrix defined by the number of terms used by a particular observer and the number of landscapes in a study (10 for the Devon studies and 11 for the Asturias study). An observer’s terms were specified in the first row, and the different landscape sites in the first column, with scores for each landscape on each term filling the resulting data matrix.

### Computation of Consensus Parameters

The statistical procedures described in this section consist of a complex series of calculations. These were integrated into a single programme by Dr. E.A. Hunter of Biomathematics and Statistics Scotland. This programme can, with some training, be run by statistical non-experts and can be obtained without cost from Prof. Francoise Wemelsfelder.

The concordance between observer matrices in each study was investigated using a multivariate statistical technique called Generalized Procrustes Analysis (GPA) [19]. GPA does not depend on the use of fixed variables, and can be thought of as a pattern matching mechanism, assuming that even if observers use different variables (terms) for measurement, the distances between measured units (landscapes) will be comparable because these units are the same. Geometrically, the collection of scores given to a landscape determines the location of a point in a multidimensional real space, with as many dimensions as the number of terms evaluated. Hence, each data matrix can be regarded as representing a geometrical configuration or arrangement of landscapes in such a space according to their scores. Equi-dimensionality across data matrices is achieved by adding columns of zeros to individual matrices to match the matrix with the largest number of terms. Configurations of landscapes from different observers are then matched as closely as possible through a series of iterative matrix transformations (translation, rotation/reflection and scaling) aimed at minimizing the sum of the squared distances between the same units for different observers, while maintaining the relative inter-unit distances within each data matrix. Once no improvement in this criterion is gained by further transformations, the average matrix of the individual transformed data matrices is taken as the ‘consensus profile’ across observers. The representativeness of the consensus profile is quantified by the Procrustes Statistic (PS), which gives the percentage of the total variance between observer configurations explained by the consensus configuration (see Wemelsfelder et al. [23] for a more detailed explanation of GPA computation steps). A consensus profile was calculated for each of the three landscape studies.

The significance of these consensus profiles was evaluated using a randomization test. Original observer data matrices in each study were analyzed in randomized form 100 times, and mean and standard deviation of the ensuing 100 PS values were calculated to reflect a random association between matrices for each study. A 1-tailed Student-t-test (n = 100, df = 99) was used to determine whether the consensus PS differed significantly from this randomized PS. A probability of p<0.001 was taken to indicate that the consensus profile was a meaningful feature of the data set and not a statistical artefact. Principal Coordinate Analysis (PCO) of PS values for all possible pairs of observers (i.e. the distances between transformed observer configurations) allows mapping observers onto a 2-dimensional observer plot. Using robust methods (i.e. not influenced by outliers), PCO estimates the centre of distributions of observers together with a standard deviation, and draws a 95% confidence region. Observers lying outside this region are potentially outliers, and possible reasons for their greater distance from the consensus can be considered. GPA can then be re-run without these observers to investigate whether and how their data affected the consensus profile.

### Interpretation of Consensus Profiles

GPA transforms individual observer configurations into one multidimensional consensus profile, independently of any interpretative judgment of observers’ terms. This consensus profile is defined in terms of its geometrical properties and has no semantic connotations attached to it. A first step towards interpretation is to determine the main dimensions of the consensus profile that explain most of the variation through Principal Component Analysis (PCA). This produces one or more 2-dimensional ‘landscape plots’ with a standard-error ellipse indicating the reliability of each landscape’s position on the main consensus dimensions. The second step is to confer semantic meaning on to those dimensions by correlating their coordinates with those of each of the original observer data matrices. This analysis results in two-dimensional ‘word charts’ (1 for each observer). In each chart, all terms of a particular observer are correlated to the first 2 (or 3rd and 4th) principal dimensions of the consensus profile. The higher a term’s correlation with a dimension, the more weight it has as a descriptor for that dimension.

Comparison of these word charts is as important a measure of agreement as the Procrustes Statistic. The question is whether meaningful semantic concurrence can be detected between individual observer word charts in their alignment of descriptors along consensus dimensions. In principle it is possible to find a significant consensus profile which semantically makes little sense. However if alignment of terms across observer word charts does make sense, a third and final step of interpretation is for the experimenter to summarize this information into one or more labels for the main consensus dimensions. This interpretative process is entirely ‘post-hoc’ and plays no role in the computation of the consensus profile. The strength of GPA is that it preserves semantic information as part of the analysis of data sets, independently of the experimenter’s interpretation of that information. This makes it possible to investigate whether or not observers apply their qualitative vocabularies in similar ways, to characterize landscapes, animals, or other subjects of attention. The terms selected as labels for consensus dimensions must be as representative as possible for the pool of strongly correlating terms associated with those dimensions, and tend to include terms used by more than one observer. The final selection of dimension labels will inevitably involve some form of judgment by the experimenter, and so to keep this judgment in perspective and avoid unwarranted reduction of perceived expressivity, labels should always be considered against the background of the entire pool of high-loading terms.

### Comparison of Consensus Profiles in Devon Outdoor and Indoor Studies

To compare DOS and DIS assessments of the 10 selected Devon landscapes, landscape scores on the main DOS and DIS consensus dimensions were correlated using Spearman Rank correlations. Spearman rather than Pearson correlations were used because scores on DOS dimension 2 were not distributed normally.

## Results

### Consensus Parameters

Table 3 shows the consensus parameters for the Devon and Asturias studies, indicating that observer assessments in each study showed significantly higher concordance than could be explained by random association. Inspection of observer plots showed there were no outliers in any of the three studies, so no further analysis was necessary.

**Table 3:** Procrustes Statistics for Devon outdoor, Devon indoor and Asturias studies.

Landscape Study	Consensus Procrustes Statistic	Randomised Procrustes Statistic ± SD	Student t df = 99	p value
**Devon Outdoor**	84	64 ± 1.03	19.33	***
**Devon Indoor**	82	73 ± 0.64	13.99	***
**Asturias**	78	71 ± 0.42	16.42	***

*** p<0.001

Table 4 shows the percentage of variation between landscapes accounted for by the first three consensus dimensions in each analysis, indicating that the first two dimensions absorb most of the variation and are worth investigating further.

**Table 4:** Percentage of variation in consensus profile accounted for by GPA dimensions 1–3.

Landscape Study	Consensus Dimensions		
	1	2	3
**Devon Outdoor**	60.7	10.8	7.0
**Devon Indoor**	44.8	12.6	8.9
**Asturias**	29.0	23.2	9.5

### Interpretation of Consensus Profiles

**Fig 1:**
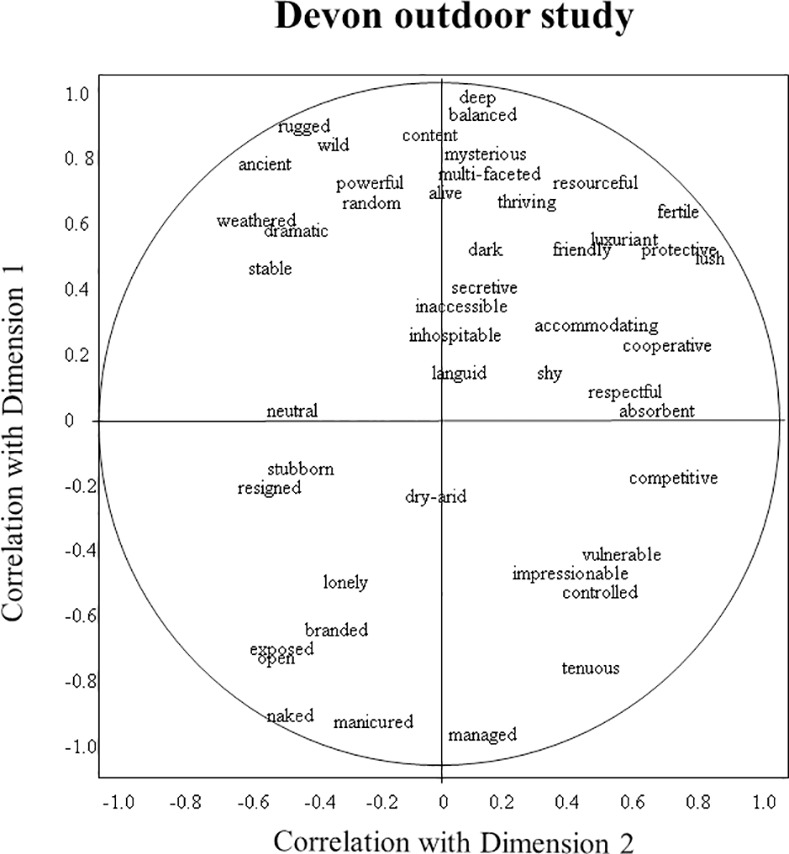
Example observer word chart of the Devon outdoor study. Axes reflect the correlation of an observer’s terms with dimensions 1 and 2 of the consensus profile.

**Fig 2:**
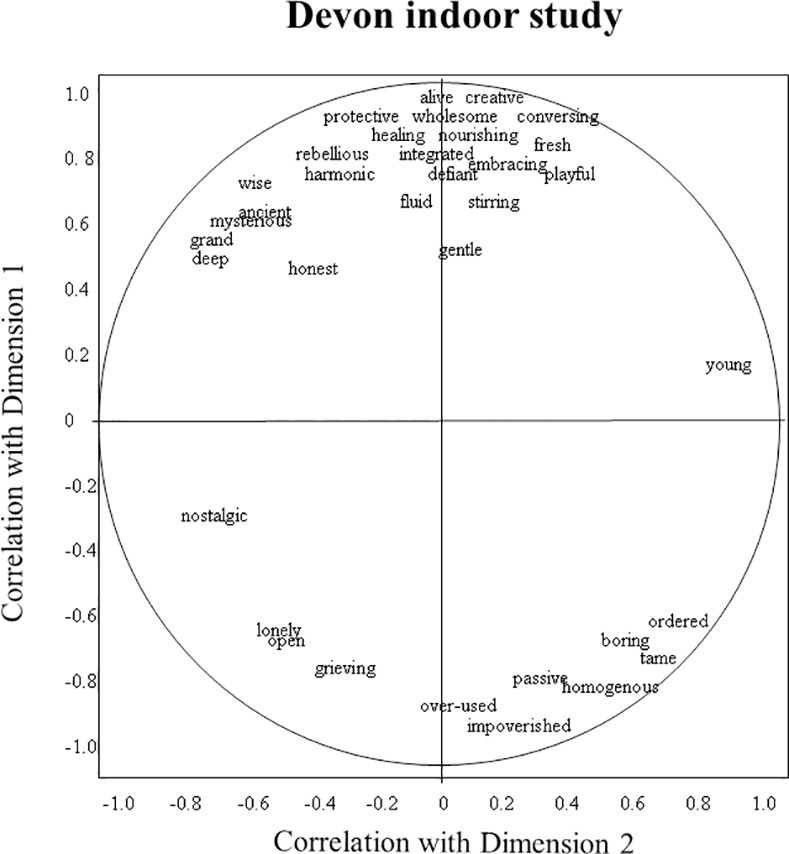
Example observer word chart of the Devon indoor study. Axes reflect the correlation of an observer’s terms with dimensions 1 and 2 of the consensus profile.

**Fig 3:**
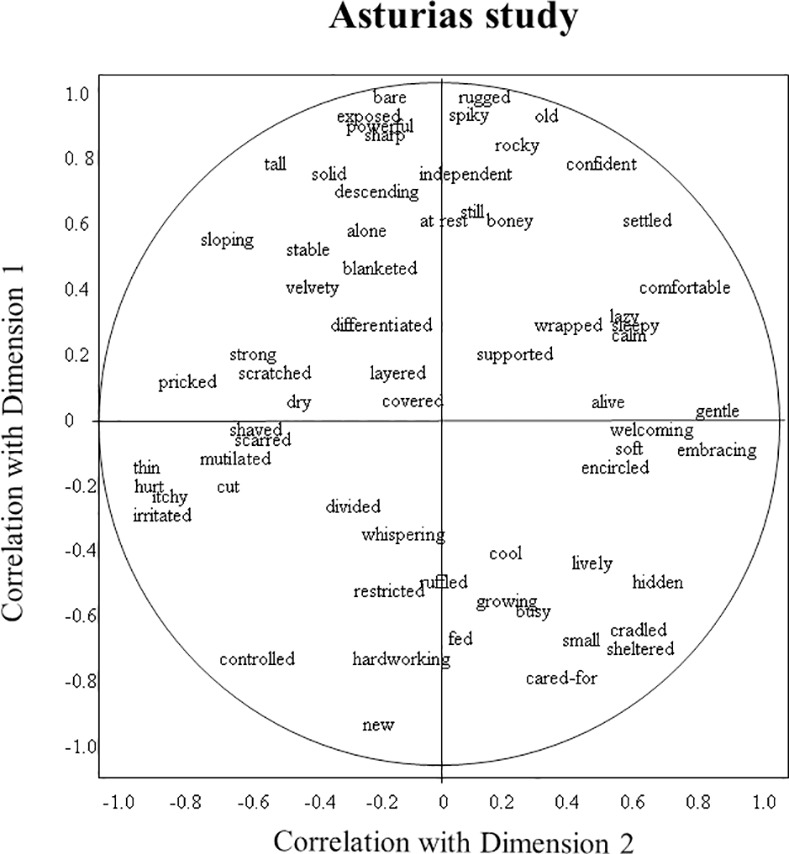
Example observer word chart of the Asturias study. Axes reflect the correlation of an observer’s terms with dimensions 1 and 2 of the consensus profile.

Figs 1–3 show as examples the word charts of one DOS, DIS, and AS observer. The DOS observer characterised dimension 1 as ranging from ‘deep/balanced/content’ to ‘managed/manicured/naked’, while the DIS observer described it as ranging from ‘creative/alive/wholesome’ to ‘impoverished/homogenous/over-used’. The DOS observer perceived dimension 2 as ranging from ‘lush/protective/competitive’ to ‘exposed/weathered/resigned’, while the DIS observer saw it as ranging from ‘young/ordered/tame’ to ‘nostalgic/deep/grand’. The AS observer described dimension 1 as ‘rugged/bare/old’ versus ‘new/cared-for/controlled’, and dimension 2 as ‘gentle/embracing/welcoming’ versus ‘irritated/hurt/thin’.

However to interpret the outcomes of this study, all observers’ assessments must collectively be taken into account. To allow comparison of observer word charts, Table 5 gives an overview of the highest correlating terms with dimensions 1 and 2 of the DOS, DIS and AS consensus profiles. It should be noted that in multivariate analysis it is arbitrary which terms end up at either the positive or negative pole of a dimensional construct, so negatively valenced terms such as bored, depleted and tired could equally have been placed at the positive end of Dimension 1, and positively valenced terms (e.g. harmonious, balanced) at the negative end. However for the sake of common sense ease of use, we chose to place positively valenced terms at the positive end of dimensions 1 and 2, and negatively valenced terms at the negative end. This association continues in the remainder of this paper, however it should be clear that this is not due to some intrinsic linguistic sensitivity of multivariate analysis.

**Table 5:** Terms (2 for each observer) showing the highest positive and negative correlations with Dimensions 1 and 2 of the consensus profile in each study. Values in brackets give the number of times a term occurs, unless occurring once.

**Devon Outdoor Study**	
**Positive correlation with Dimension 1**	**Negative correlation with Dimension 1**
Harmonious, balanced, grounded, authentic, wise, deep, pure, ancient, sedate, magical, secretive, playful, happy, energised, dynamic, eloquent, plentiful, diverse, lively, satisfied	Bored (4), depleted, tired, numb, subdued, dead, violated, frustrated, restrained, conditioned, naked, managed, manicured, maintained, exposed, waiting, longing
**Positive correlation with Dimension 2**	**Negative correlation with Dimension 2**
Lush (2), lustrous, vibrant, fresh, moist, life-giving, adventurous, boisterous, fertile, diverse, sheltered, protective, nested, fragile, soft, forgiving, loving, feminine, lazy	Exposed (2), weathered (2), solid (2), massive, resilient, unshakable, hardened, harsh, eroded, masculine, naked, forgotten, patient, quiet, calm, fair, peaceful
**Devon Indoor Study**	
**Positive correlation with Dimension 1**	**Negative correlation with Dimension 1**
Alive (3), playful (2), harmonious, natural, wild, energetic, dynamic, creative, excited, enthusiastic, light-hearted, bright, meaningful, connected, abundance, green-depth, restful	Depleted (2), tired, impoverished, barren, misused, damaged, long-suffering, resigned, accepting, regimented, hard-working, homogenous, dry, windswept, open, rolling, sharp
**Positive correlation with Dimension 2**	**Negative correlation with Dimension 2**
Noisy (2), fresh, aspiring, young, newness, to-play, invitation, supportive, smiling, honest, gentle, quiet, sad, sadness, captive, resigned, obedient, constrained, ordered	Deep (2), bare, wind-blown, a-picnic-spot, strong, latent-power, stately, abiding, knowing, surviving, silence, sombre, lonely, nostalgic, secretive, bored, tired, accusing, strife
**Asturias Study**	
**Positive correlation with Dimension 1**	**Negative correlation with Dimension 1**
Solid (2), immovable (2), solitary (2), surly (2), imposing, superb, authoritarian, strength, dangerous, character, longevity, confident, secure, ambitious, mature, experience, wise, rugged, bare, naked, exposed, cutting, aloof, observer	Ordered (2), cared-for (2), imposed-upon, forced, modelled, managed, manicured, suppressed, anthropocentric, nurtured, balanced, tolerant, complacent, conformist, docile, tranquil, shadowed, humble, careful, new, youthful, stranger, vital, extrovert, sociable, affectionate
**Positive correlation with Dimension 2**	**Negative correlation with Dimension 2**
Welcoming (2), inviting, embracing, cradling, accommodating, conformist, integration, versatile, captivating, intense, wild, inspiring, harmony, playful, active, friendly, loyal, nutritious, beauty, happy, joy, relaxed, gentle, calm, peaceful, sluggish, sensible	Organised (2), tired (2), hurt (2), sad (2), disciplined, strong, competitive, voracious, extreme, arrogant, resigned, passive, impaled, suffocated, denatured, infiltrated, helpless, twisted, irritated, confused, scared, poor, thin, alien

From Table 5 it appears that there is considerable semantic convergence between terms used to describe the positive and negative ends of DOS, DIS, and AS consensus dimensions. Terms at either end of a dimension seem to indicate complementary aspects of landscape ‘atmosphere’ (e.g. harmonious, balanced, grounded in DOS1, or alive, energetic and bright in DIS1), particularly when considered in contrast to the expressive qualities at the opposite end of the dimension.

In the Devon studies the first dimension appears to contrast harmonious, alive, abundant, happy landscapes with depleted, numb, regimented, bored ones. The added presence of terms such as wild, pure, natural and authentic on the positive end, and manicured, violated and misused on the negative end, indicates that observers associated this contrast with a detrimental effect of human intervention. The second dimension further differentiates the qualities of dimension 1, distinguishing fresh, new, and fragile, from weathered, eroded, and resilient landscapes. This contrast can apply to both ends of dimension 1, and terms for dimension 2 can therefore depict a mixture of positive and negative atmospheres. As Table 5 shows for DIS for example, a new or young landscape (positive side of dimension 2) can be fresh and inviting (on the positive side of dimension 1), but when misused and depleted (on the negative side of dimension 1) can also be sad and resigned; a bare, wind-blown landscape (negative side of dimension 2) can be strong and stately (on the positive side of dimension 1), but when misused and depleted (on the negative side of dimension 1) can also be sombre and lonely. This differentiation between positive and negative aspects of dimension 2 is also reflected in the DOS and DIS word chart examples.

In the Asturias study, the first two dimensions are more equal in strength than they are in the Devon studies, with the first dimension much weaker and the second dimension twice as strong as in the other studies. The first dimension appears to contrast immovable, rugged, imposing, confident landscapes with ordered, youthful, docile, tranquil landscapes. Here too, the association with human intervention is evident in terms such as cared-for, imposed-upon, anthropocentric and nurtured, but in contrast to the Devon studies, this intervention seems not to necessarily be perceived in detrimental terms. The second dimension further differentiates the qualities of dimension 1, distinguishing welcoming, accommodating, friendly places from organised, resigned, sad places. Here, the presence of terms such as impaled, suffocated, denatured, infiltrated, helpless, and twisted on the negative side suggests that this dimension does primarily serve to differentiate beneficial from detrimental human influence.

**Fig 4:**
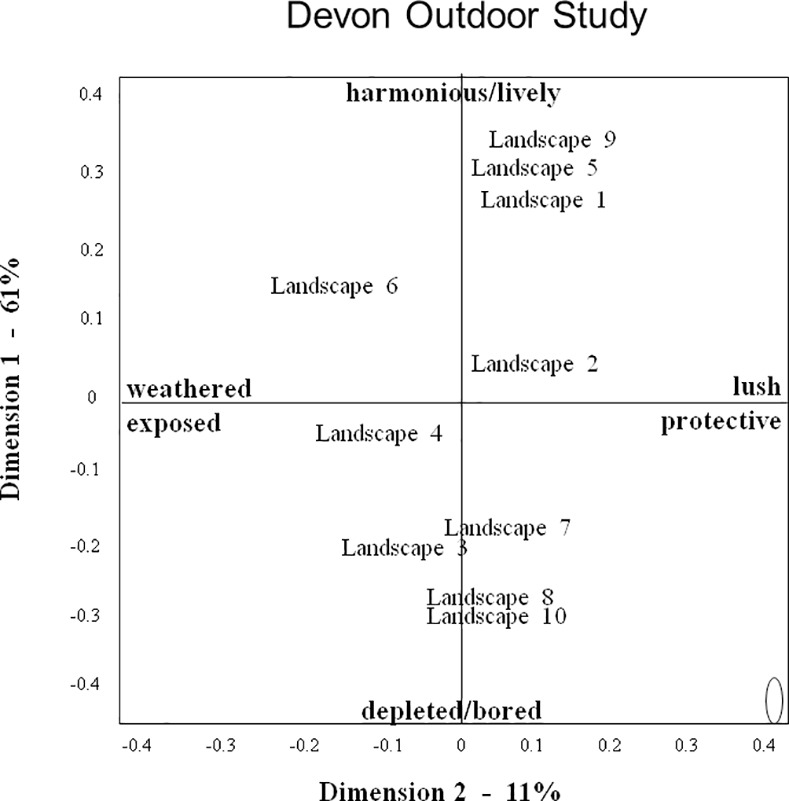
Landscape Plot for the Devon outdoor study. Axes reflect scaling-values for the relative distance between landscapes on dimensions 1 and 2 of the consensus profile. The ellipse represents the standard error for each landscape’s position in the plot.

**Fig 5:**
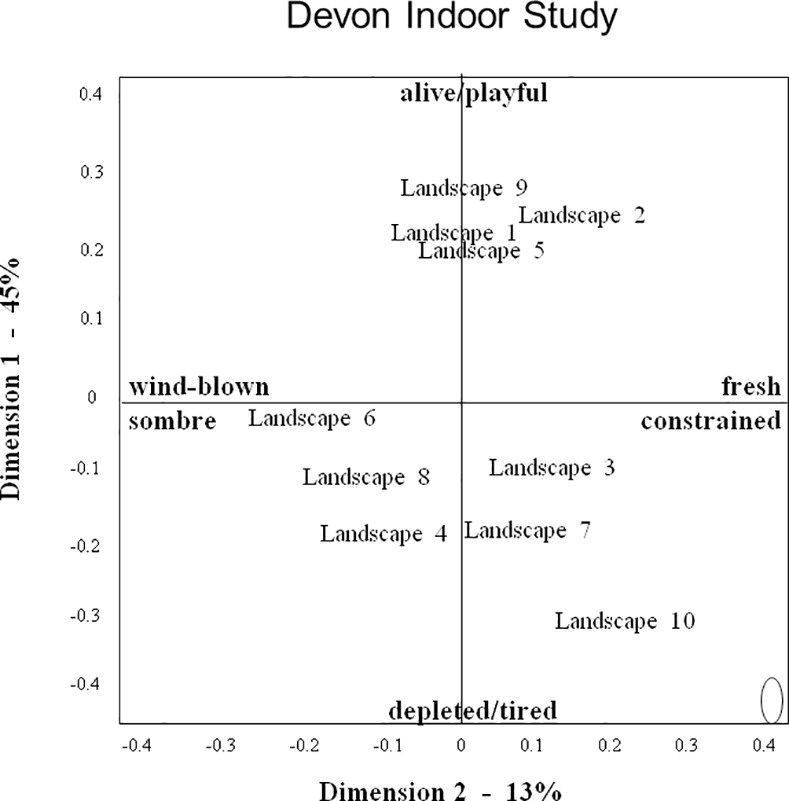
Landscape Plot for the Devon indoor study. Axes reflect scaling-values for the relative distance between landscapes on dimensions 1 and 2 of the consensus profile. The ellipse represents the standard error for each landscape’s position in the plot.

**Fig 6:**
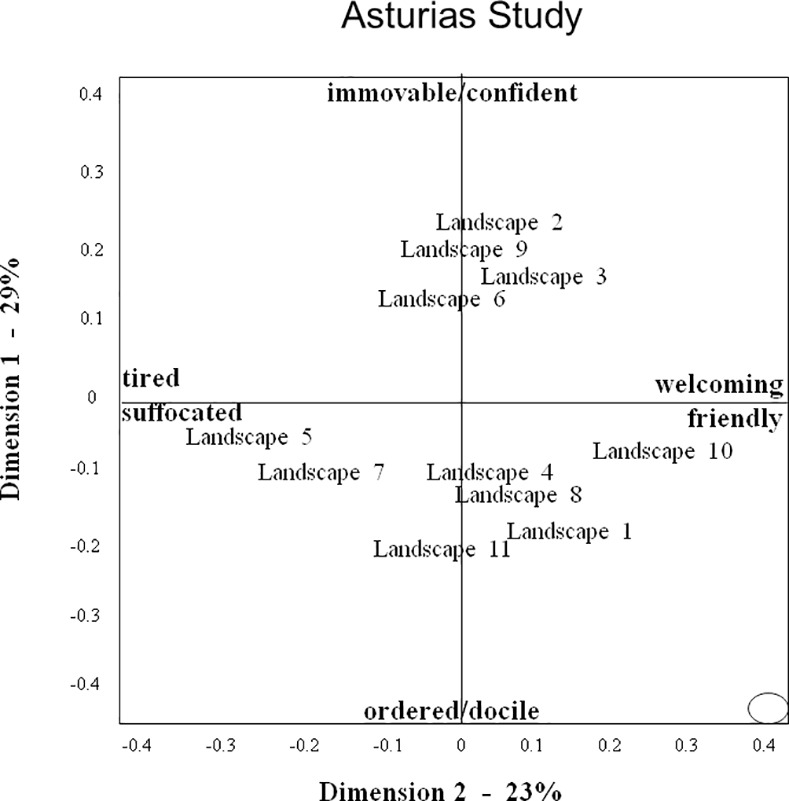
Landscape Plot for the Asturias study. Axes reflect scaling-values for the relative distance between landscapes on dimensions 1 and 2 of the consensus profile. The ellipse represents the standard error for each landscape’s position in the plot.

Thus observers collectively created a complex judgment of landscape expressivity, in which their many terms converge to indicate certain themes, yet within these themes also vary in tone and focus. The variety and subtlety of perceived qualitative atmospheres makes it difficult to summarise the information presented in Table 5. However for the benefit of further discussion, it is useful to select a number of terms as interpretative labels for the various dimensions (for criteria see Methods), while acknowledging the restrictive nature of such labels. We suggest the following labels for the consensus dimensions of the three studies: DOS dimension 1: harmonious/lively–depleted/bored; DOS2: lush/protective–weathered/exposed; DIS1: alive/playful–depleted/tired; DIS2: fresh/constrained–windblown/sombre; AS1: immovable/confident–ordered/docile; AS2: welcoming/friendly–tired/suffocated. We selected these labels because we considered them to suitably reflect the various qualities associated with the different dimensions, however they should not be regarded as final interpretations, but more as ‘working summaries’ that can help interpret the information presented in Figs 4–6.

### Landscape Characterisations

Figs 4–6 show the ‘landscape plots’ for the three studies. In all studies the standard error of the coordinates of individual landscapes is small (as indicated by the ellipse in the bottom right hand corner of each figure), and so these coordinates can be assumed to reliably characterize the landscapes’ position on dimensions 1 and 2.

Landscape scores on DOS and DIS dimension 1 were significantly correlated (r = 0.82, p<0.004). In both DOS and DIS, the highest positive scores on dimension 1 (DOS1: harmonious/lively; DIS1: alive/playful)) were attributed to pristine riverine landscapes surrounded by native woodland (landscapes 9, 5, and 1). In DIS a semi-natural but undisturbed hillside oak wood (2) was also included in this cluster, but DOS placed this wood somewhat further down towards the ‘depleted/bored’ end. DOS placed the Dartmoor Tor rocks (6) on the positive side just below the riverine landscapes, whereas DIS put it on the negative side (depleted/tired) nearer to the man-made reservoir (8) and heathland (3). In both DOS and DIS the lowest negative score on dimension 1 (DOS1: depleted/bored; DIS1: depleted/tired) was attributed to a large plowed agricultural field (10), closely followed in DOS by a man-made reservoir (8). DIS ranked this reservoir more positively than a high moor (4) and scattered fields and woods (7), however DOS and DIS assessments agreed in placing these landscapes on the negative side of dimension 1.

Landscape scores on DOS and DIS dimensions 2 were not significantly correlated (r = 0.42, NS). Both DOS and DIS attributed their most negative scores on dimension 2 (DOS2: weathered/exposed; DIS2: wind-blown/sombre) to Dartmoor Tor rocks (6) and the high moor (4), but they differed in how they ranked landscapes at the positive end. DOS gave highest positive scores on dimension 2 (lush/protective) to riverine landscapes located on the positive side of dimension 1, whereas DIS gave the agricultural field (10), located at the negative side of dimension 1, a considerably higher positive score on dimension 2 (fresh/constrained) than other landscapes. Associated with this difference, the positive end of DOS dimension 2 is mainly characterized by positive terms such as lush and vibrant, whereas the positive end of DIS dimension 2 also contains a number of negative terms pertaining to the field, such as sad, resigned and constrained. By contrast, DIS placed the man-made reservoir (8) considerably further to the negative side of dimension 2 than DOS, characterizing it as wind-blown and sombre rather than fresh and constrained.

In AS, the landscape plot presents a somewhat triangular picture, with only landscapes positioned on the negative side of dimension 1 showing a differentiation on dimension 2. On dimension 1, much weaker than in the Devon studies, there are basically two clusters of landscapes: extended mountainous landscapes (2,3,6,9) on the positive side (immovable/confident), and all other landscapes on the negative side (ordered/docile), including a razed mountain scarred by a road (5), a eucalyptus forestry plantation (7), semi-natural grazing pastures (8,10), a village in mature woodland (4), and ornamental and vegetable gardens (1,11). In the latter group, dimension 2 (twice as strong as in the Devon studies) differentiates between the razed mountain (5) and forestry plantation (7) on the negative side (tired/suffocated), and a small grazing pasture surrounded by natural woodland (10) on the positive side (welcoming/friendly), with the other landscapes arranged between the two.

## Discussion

The results of this study demonstrate that three independent groups of observers, using a Free Choice Profiling (FCP) methodology, each showed significant agreement in how observers, using their own words, assessed the expressive qualities of Devon (UK) or Asturian (Spain) landscapes. Multivariate analysis showed that all three observer groups generated two consensus dimensions, which can be summarized as follows: Devon outdoor study dimension 1 (DOS1): harmonious/lively–depleted/bored; DOS2: lush/protective–weathered/exposed; Devon indoor study dimension 1 (DIS1): alive/playful–depleted/tired; DIS2: fresh/constrained–windblown/sombre; Asturias study dimension 1 (AS1): immovable/confident–ordered/docile; AS2: welcoming/friendly–tired/suffocated. These characterisations suggest that the two observer groups assessing the same Devon landscapes, either through personal visits (DOS) or from digital images (DIS), generated consensus dimensions painting comparable contrasts in landscape ‘atmosphere’. For the first dimension, this similarity is supported by the strong correlation between DOS1 and DIS1 landscape scores. For the second dimension however, the lack of significant correlation between DOS2 and DIS2 landscape scores suggests that despite apparent similarities in descriptive terminology (e.g. lush vs fresh, weathered vs wind-blown) the two groups in fact differed in how they characterized individual landscapes on this dimension. The consensus dimensions for the Spanish landscapes cannot be directly compared to those for the Devon landscapes, however the terms used and contrasts painted in the three studies suggest some common underlying themes in the observers’ appreciation of landscape quality.

A primary theme, as suggested by the terms associated with DOS/DIS dimensions 1 and AS dimension 2, was the loss of authenticity, vibrancy, and abundance in landscapes strongly disciplined by humans. These landscapes were perceived as tired, numb, bored, damaged, or worse, as violated, suffocated, misused–all terms that indicate ill health, but also aggressive and abusive interference. However not all human intervention was perceived to have such negative effect; on dimension 1 of the Asturias study human presence was also seen to make landscapes welcoming, inspiring and nutritious. Regardless of whether the effect was positive or negative, it is striking how prominently notions of health and well-being feature in these assessments of landscape. A second theme in all three studies (DOS/DIS dimensions 2 and AS dimension 1) was that of development in time, with some landscapes perceived as young, fresh, dynamic, boisterous, and fragile, and others as ancient, abiding, patient, wise, and resilient. This distinction does not appear to be linked to what humans do–both young and old landscapes could be seen as welcoming (‘a picnic spot’) or as suffering restraint (e.g. ‘sad’, ‘bored’).

Thus, through quantification of their personal terminologies, the three observer groups provided coherent and meaningful judgments of landscape quality, and the emerging themes appear to support our guiding hypothesis–that landscapes, as places of collective living, express sensitivity. Landscape impressed itself on observers not as inert aggregated material to be shifted around without consequence, but as a responsive presence, which, when not appropriately treated, loses its health and suffocates. The perceived temporality of landscapes also links in with this perception, in that temporality is an important dimension of embodied living [11]. Again nature is not perceived as dead stuff shifting around, but as a dynamic process connecting past, present and future–making landscapes patient, or wise. It is interesting that observers in the Devon indoor study also basically produced these same themes from still images, though explaining less data variance than observers in the outdoor study, and differing in the characterisation of some landscapes. We did of course specifically instruct observers to assess landscape expressivity, but we did not in any way refer to landscape health or temporality, and so these themes are interesting independent outcomes. They are reminiscent, for example, of the ideas of Aldo Leopold, who, in his seminal work on land ethic and stewardship, spoke of ‘land health’ and ‘land sickness’, and the need to preserve “the integrity of the biotic community” ([24], p.279).

Scientists may be inclined to disregard such qualifications of landscape as super-imposed, anthropomorphic projections, leading to undesirable personification of what they assume are blind natural processes. One only needs to think of the Gaia hypothesis controversy. However, a growing number of voices are calling on academia to allow itself the freedom and flexibility to explore different perspectives on nature and its landscapes, out of respect for other cultures, and to encourage a more participative understanding of landscape ecology and conservation [25–28,12]. The philosopher Mary Midgley is well-known for her advocacy of a pluralistic science: *“We know now that science always uses imaginative visions or paradigms which change from time to time*, *along with the imagery that expresses them*. *What matters is to grasp what the changing images mean*. *Personifying the earth means that it is not just a miscellaneous heap of resources but a self-maintaining system that acts as a whole*. *It can therefore be injured; it is vulnerable*, *capable of health or sickness*. *And*, *since we are totally dependent on it*, *we are vulnerable too”* ([29], p.8). The findings of the present study, we suggest, should be taken in this spirit–not as making any strong objectivist claims, but as exploring languages of human-nature relationship.

Within this context, an interesting question is to what extent observers succeeded, as instructed, in not letting their personal likes and dislikes dominate their perception of the landscapes. When describing landscapes as ‘inviting’, ‘accepting’, or ‘accusing’, did they feel invited, accepted, or accused, or did they locate these qualities with the landscapes? Such questions refer to the dynamics of communication and are difficult to resolve–probably both are true to some extent. Could one perceive a landscape as inviting when one would not personally want to go there–probably not. But could one perceive a landscape as bored when one was not bored oneself–obviously yes. By and large the terminologies observers generated for this study do appear intended to address the landscapes. However, this is not to say that observers’ predispositions would not have influenced their choice of terms. The observers selected for this study were, given their backgrounds, very amenable to viewing landscape psychologically, and very likely to take a critical view of industrialisation. It would be important therefore to repeat this study with observers with different backgrounds and predispositions, such as industrial farmers or urban planners. If landscape qualities had any sort of reality beyond observers’ individual aesthetic idiosyncrasies, one would expect to find at least basic levels of agreement between such different groups.

Indeed with animals this appears to be the case; pig farmers, veterinarians and animal activists, using FCP to judge the quality of pig expressions, were found to show good agreement despite their different backgrounds and attitudes [30]. Thus when observers are appropriately instructed to directly engage with animals, background differences do not necessarily lead to disagreement. The same could potentially apply to landscapes, although finding grounds for agreement may take longer. A photo-based study by Van den Berg et al. [31], for example, found that Dutch farmers saw greater beauty in agrarian, cultivated landscapes than in wilder, less cultivated landscapes, and also scored these landscapes more highly in terms of their complexity, coherence, mystery and diversity. By contrast, non-farming residents and visiting cyclists found the wilder landscapes more beautiful, and attributed these landscapes with greater complexity, coherence, mystery and diversity. Thus farmers and non-farmers evaluated given landscape attributes in very different ways, aligning their ratings of these with their aesthetic preferences. Crucially however, the Van den Berg study was set-up as a study of aesthetic evaluation, presenting participants with pre-defined questions and attributes to be imposed *on* the landscapes. This is very different from the current study, which asked participants to discern meaning *from* the landscapes. But even then, industrial farmers could quite feasibly perceive positive expressive qualities in agrarian landscapes that non-farmers might not recognise.

We don’t think, however, that such disparities, if they occur, should immediately throw us back on to the subjectivist/objectivist dichotomy. The key question at this early stage of investigation is not whether observers agree perfectly, as a necessary condition for accepting landscape expressivity as ‘real’, but whether or not observers are generally capable of engaging with landscapes and meaningfully read their qualities, and learn from these. Qualities are not fixed, static objects looking exactly the same from all angles. They are fluid, layered, context-dependent communications, and people may, in the first instance, communicate differently with the same landscapes, and so appear to see different things. But such differences are not necessarily mutually exclusive, and may enrich each other when handled well, and ultimately lead to a more complex and inclusive consensus. As Van den Berg and colleagues [31] acknowledge at the end of their study, it is good to admit and investigate difference, but it is undesirable to then reduce this difference to a right/wrong (objectivist/subjectivist) model. This would quite fundamentally obstruct communication between stakeholder groups, and would not serve an inclusive approach to ecological and cultural sustainability [27].

The larger goal, then, is to explore open-mindedly the merits of people’s assessments of landscape expressivity. The present study has found that participants, if and when appropriately instructed, appear to be able to make such assessments in coherent and meaningful ways. The strength of FCP methodology is that it lets observers make spontaneous judgments and preserves this information, yet combines this with quantitative scoring and statistical analysis, to generate expressive scores that can be used in further scientific analysis [20]. This strength can be applied in different ways. One of these is to link qualitative landscape assessments with quantitative measures of ecological biodiversity and sustainability. It would be useful to know whether and how the landscape terminologies of different observer groups (from lay people to professional ecologists) correlate with indices of biodiversity in different habitats. If this were the case, then such terminologies could potentially contribute to context-sensitive assessments of ecosystem health, and facilitate the interpretation of quantitative ecological indices. Such applications are a long way off, but it is perhaps good to acknowledge that experienced ecologists, when pressed, will often admit to already using qualitative judgments in assessments of ecosystem health. Formal recognition and analysis of such perceptions could stimulate our sensitivity to landscapes, and open up new areas of research.

## Supporting Information

S1 File Observer FCP scores for the Devon Outdoor Study. (XLSX)

S2 File Observer FCP scores for the Devon Indoor Study. (XLSX)

S3 File Observer FCP scores for the Asturias Study. (XLSX)

S4 File English translation of Spanish observer terms. (XLSX)
